# A phase II study of Yondelis® (trabectedin, ET-743) as a 24-h continuous intravenous infusion in pretreated advanced breast cancer

**DOI:** 10.1038/sj.bjc.6603142

**Published:** 2006-05-30

**Authors:** L Zelek, A Yovine, E Brain, F Turpin, A Taamma, M Riofrio, M Spielmann, J Jimeno, J L Misset

**Affiliations:** 1 Department of Medicine, Institut Gustave-Roussy, Villejuif, France; 2 Department of Medical Oncology, Henri Mondor Hospital, Créteil Cedex, France; 3 Service d'Oncologie, Hôpital Paul-Brousse, Villejuif, France; 4 Department of Medicine, Centre René-Huguenin, Saint-Cloud, France; 5 Cvitkovic and Associates, Kremlin-Bicêtre, France; 6 PharmaMar R&D, Madrid, Spain

**Keywords:** Yondelis, trabectedin, breast cancer

## Abstract

Yondelis® (trabectedin, ET-743) is a novel marine-derived anticancer compound found in the ascidian *Ecteinascidia turbinata*. It is currently under phase II/III development in breast cancer, hormone refractory prostate cancer, sarcomas and ovarian cancer. Activity in breast cancer experimental models has been reported, and preliminary evidence of activity in this setting during the phase I programme has also been observed. The present study assessed the activity and feasibility of trabectedin in women with advanced breast cancer previously treated with conventional therapies. Patients with advanced disease previously treated with at least one but not more than two regimens that included taxanes or anthracyclines as palliative therapy were eligible. Trabectedin 1.5 mg m^−2^ was administered as a 24-h continuous infusion every 3 weeks. Patients were kept on therapy until disease progression, unacceptable toxicity or patient refusal. Twenty-seven patients were included between April 1999 and September 2000. Their median age was 54 years (range: 36–67) and 63% of them had two metastatic sites. Twenty-two patients were performance status 1. All patients had previously received anthracyclines, and 23 out of 27 patients had received taxanes. Of 21 patients with measurable disease, three confirmed partial responses, one unconfirmed partial response and two minor responses (49 and 32% tumour shrinkage) were observed; six patients had stable disease. Median survival was 10 months (95% confidence interval: 4.88–15.18). Transient and noncumulative transaminitis was observed in most of the patients. The pharmacokinetic profile of trabectedin in this patient's population is in line with the overall data available with this schedule. The policy of dose adjustments based on the intercycle peaks of bilirubin and alkaline phosphatase appears to have a positive impact in the therapeutic index of trabectedin. Trabectedin can induce response and tumour control in previously treated advanced breast cancer, with manageable toxicity, thus warranting further development as a single agent or in combination regimens.

Breast cancer is a chemosensitive disease, and systemic approaches as neoadjuvant or adjuvant therapy can have a significant impact on the disease (Crown, 2004; Pegram, 2004). The role of chemotherapy in the advanced or metastatic setting, however, is palliative, and novel approaches with taxanes, new vinca derivatives, new antimetabolites, well-designed combination strategies and targeted therapies (Hortobagyi, 2003) have been successfully developed. Nevertheless, long-lasting responses are rare, and the identification of new drugs that are active in this setting remains a challenge.

Trabectedin is a marine-derived DNA-interacting agent and transcription inhibitor (D'Incalci and Jimeno 2003; Martinez *et al*, 2005) found in the ascidian *Ecteinascidia turbinata* (Figure 1). It is currently under phase II/III development in advanced breast, hormone refractory prostate, pretreated soft tissue sarcoma and relapsed ovarian cancer. *In vitro* and *in vivo* activity have been reported in experimental breast cancer models, including evidence of a complete absence of cross-resistance with doxorubicin and paclitaxel and a positive *in vivo* therapeutic index in the MX-1 doxorubicin-resistant model (Jimeno *et al*, 1996). In a phase I study using a 24-h intravenous (i.v.) continuous infusion (Taamma *et al*, 2001), the maximum tolerated dose was 1.8 mg m^−2^, with pancytopenia and fatigue as dose-limiting toxicities. At the recommended dose of 1.5 mg m^−2^ every 3 weeks, early onset, reversible and noncumulative transaminitis was observed in the majority of 25 patients included at this dose level. Twenty per cent of grade 4 neutropenia was noted in this heavily pretreated population, and the incidence of thrombocytopenia was low. Drug-induced emesis could be controlled by conventional supportive procedures, and there was no alopecia, mucositis, diarrhoea or other symptomatic toxicities. Results at this recommended dose level showed no cumulative toxicities. Furthermore, a partial response was observed in a previously irradiated area in a taxane-resistant patient. Based on these promising findings, we conducted a phase II study of trabectedin in patients with advanced breast cancer who were resistant or relapsed after conventional chemotherapy.

## PATIENTS AND METHODS

This multicentre, open-label, phase II nonrandomised study included three French centres. The Ethics Committee of Bicêtre Hospital, France approved the study, and all patients gave their signed informed consent. The trial was conducted according to the ICH guidelines and was partially supported by the European Commission through a BIOMED II demonstration programme (Contract no. BMH4-CT98-3614 (DG 12-SSMI)).

### Patient population

Patients with progressive advanced breast cancer previously treated with anthracyclines and/or taxanes were eligible for this study. No more than two previous regimens for metastatic disease were allowed. Four weeks had to have elapsed between completion of prior therapy and entry into the study (6 weeks for nitrosoureas). Normal cardiac, renal and bone marrow functions were required. Patients with abnormal liver function (serum bilirubin and alkaline phosphatase <ULN; ASAT and ALAT⩾2.5 × ULN) and patients with >50% liver involvement were not eligible.

### Treatment plan and dose modification

Trabectedin 1.5 mg m^−2^ was administered as a 24-h i.v. continuous infusion, with appropriate antiemetic medication, every 3 weeks. Haematological growth factors were not routinely administered. The dose was reduced to 1.2 mg m^−2^ in case of grade 4 neutropenia lasting >5 days, neutropenic fever, or grade 4 thrombocytopenia and to 1 mg m^−2^ in case of further bone marrow toxicity. Treatment was delayed for a maximum of 2 weeks if liver and bone marrow parameters fell below acceptable levels. No dose reduction was initially planned for reversible noncumulative transaminitis. However, in the early stages of a phase II programme, the incidence of severe multiorgan toxicities leading to rabdomyolisis and renal failure was of concern, prompting the investigators to conduct a pharmacokinetic (PK)/pharmacodynamic analysis, the results of which demonstrated the impact of biliary function at baseline and the clinical relevance of abnormal intercycle peaks of bilirubin and alkaline posphatase (Gómez *et al*, 2000) as risk factors for serious toxicities. Based on these findings, the protocol was amended after the first 14 patients had been entered, and a procedure on dose reduction related to liver toxicity was included: grade 3–4 transaminitis still present at day 21 but reversible by day 28 required a dose reduction to 1.2 mg m^−2^, with a subsequent dose reduction to 1 mg m^−2^ permitted if necessary. An identical dose reduction policy was also mandatory in case of grade ⩾1 bilirubin or alkaline phosphatase intercycle peaks. In addition, bilirubin and alkaline phosphatase values had to be within normal values before the administration of the following cycle. Treatment was to be continued until disease progression, unmanageable toxicity or patient refusal.

### Response and toxicity assessment

Tumour assessment for all lesions was performed at least every two cycles. Any evidence of response was confirmed 4 weeks later. Response was evaluated according to the WHO criteria. A haemogram was performed at days 8, 15 and 22, and liver chemistry at days 4, 8, 15 and 22 of each cycle. A biochemical evaluation, including liver and renal function, was performed before each treatment cycle.

### Pharmacokinetic assessment

On the basis of phase I PK findings (Van Kesteren *et al*, 2000), a limited sampling method was proposed. The bioanalysis of trabectedin in plasma was performed using a miniaturised liquid chromatography coupled to an electrospray ionisation sample inlet (turbolon Spray and two mass analysers). Solid-phase extraction was used to characterise the pretreatment sample. The low limit of quantitation of the assay is 0.01 ng ml^−1^. Eight millilitres of peripheral blood were collected at baseline, at 6 h after the start of the infusion, at 30 min before the end of the infusion and at 3, 24 and 48 h after the completion of the infusion. Plasma concentration *vs* time profile was assessed for the set of PK variables defined in the phase I study (Van Kesteren *et al*, 2000): area under the curve, maximum plasma concentration, elimination half-life, total plasma clearance and volume of distribution at steady state. As early clinical data indicated a cycle-related tolerance to the trabectedin-induced hepatobiliary events, the current study includes, whenever it was feasible, a PK analysis on first and second cycles.

### Statistical analysis

The study design was based on a two-stage Gehan method to determine response rate, with a standard error <0.10. The primary end point was tumour response; secondary end points were safety, duration of response, time to progression and survival. Duration of response was calculated from the date the response was recorded to the date of progression. Time to progression and survival were measured from the first day of study treatment to the day of progression or death (or last follow-up) and analysed according to the Kaplan–Meier method.

## RESULTS

### Patient characteristics

Twenty-seven women with breast cancer from three French institutions were included. Median age was 54 years (range: 36–67) and 63% had two metastatic sites. Twenty-three patients (85%) had previously received anthracyclines or taxanes. Twenty-two patients were symptomatic at entry. Other patient characteristics are shown in Table 1.

All 27 patients were treated and considered evaluable for safety. Twenty-two patients (81.5%) were evaluable for response. Reasons for nonevaluability were: nonmeasurable lesions (four) and metastatic lesions from a controlateral breast sarcoma (one). This latter patient was considered noneligible and excluded from all efficacy analyses, but the other four patients were included in the time-to-event analysis (progression and survival). Treatment was discontinued in one patient owing to toxicity; in another patient, an unrelated serious adverse event led to patient withdrawal. A total of 104 cycles were administered. The median number of cycles per patient was 3 (range: 1–14). Four patients were treated with six or more cycles of trabectedin.

### Response and survival

The overall response rate was 14% (95% confidence interval (CI): 3.5–32%). Three patients attained confirmed partial responses lasting for 3, 7.3 and 11.3 months, respectively. Drug-induced tumour shrinkage in the target measurable lesion in these three patients was 86, 76 and 100%, respectively. A fourth patient attained an unconfirmed partial response after cycle 2 but progressed on cycle 4, with no evaluation performed on cycle 3. Two patients had minor responses (31.8 and 49.4% tumour shrinkage) that lasted 5 and 6 months, respectively. Six patients had stable disease. Ten patients had progressive disease, including three with early progression. The characteristics of patients with objective response are listed in Table 2. All objective responses were observed at the second cycle of treatment.

Sixteen patients had an abnormal Ca 15.3 at entry, eight of whom experienced a median decrease of 43.7% (range 11–92%). The median time to performance status (PS) deterioration, defined as PS⩾2, calculated from the start of trabectedin therapy, was 7.3 months.

Median time to progression was 2.14 months (95% CI: 1.09–3.19 months). Median survival was 10 months (95% CI: 4.88–15.18 months). The projected 1- and 2-year survival rates are 45.5 and 28%. All deaths were due to malignant disease.

### Safety

The median actual dose intensity was 443.7 *μ*g m^−2^ week^−1^ (range: 304–500), with a median relative dose intensity of 88.7% (range: 60.8–100). After the protocol amendment on liver toxicity was implemented, median relative dose intensity was 84.4% (range: 60.8–100), compared to 97.7% (range 64.2–100) before the amendment. Eighteen patients (67%) had at least one cycle delayed, with a median of one cycle delayed per patient (range: 1–9). A total of 47 cycles were delayed, of which 15 (32%) were due to non-treatment-related causes. Twenty-seven delays (57%) were due to haematological reasons and five delays (11%) to other events. Median duration of delay was 8 days (range: 1–19) for treatment-related delays and 7 days for delays due to other causes (range: 1–14). Dose reduction was necessary in eight of the delayed cycles (17%).

Treatment was discontinued in only one patient owing to toxicity, grade 4 neutropenia. Toxicity was mainly haematological (neutropenia) and hepatic (transaminitis) (Table 3). Eighteen patients suffered fatigue grade 1 or 2 but severe fatigue was noted in seven patients. The most frequent gastrointestinal toxicities were nausea and vomiting, usually grade 1 or 2. Mild constipation occurred in 40% of patients but was mainly due to supportive medications such as opiates.

Grade 4 neutropenia occurred in one-third of the patients. Median time to onset of grade 3–4 neutropenia was 13 days after the infusion. The median duration of grade 4 neutropenia was 6 days. No episodes of neutropenic fever were observed. Almost all patients experienced an increase of transaminases >upper normal limit (UNL), and 75% had elevated alkaline phosphatase. Transaminitis was transient, reversible and noncumulative, with an early onset, reaching a peak at a median of 5 days. Whereas 75% of patients had grade 3–4 ASAT/ALAT increases (>5 × ULN), all increases in alkaline phosphatase were grade 1–2 (<5 × ULN), and bilirubin was elevated in only three patients. The median duration of grade 3–4 cytolysis was 6 and 8 days, respectively, and median time to recovery to grade 1 (2.5 × ULN) was 15 and 16 days for ASAT and ALAT, respectively. Alkaline phosphatase peaked later than liver enzymes. The median duration of hyperbilirubinaemia was 6 days. All drug-related toxicities were noncumulative, thus making it possible to administer multiple cycles.

### Impact of the protocol amendment in the therapeutic index of Yondelis

In our study, 14 out of the 27 patients were included after the protocol amendment. The median number of cycles were three in both groups of patients. The median dose intensity (mg m^−2^ week^−1^) was 0.488 and 0.422 before and after the amendment, respectively. The cohort of patients treated after the amendment experienced a less frequency of severe neutropenia (57%) than the ones treated before the amendment (77%).

No patients treated after the amendment had treatment-related severe fatigue but such clinically important side effect was observed in four out of 13 cases treated before the implementation of the dose reduction policy (Fisher's test; *P*=0.04). One confirmed partial response was reported among the cases treated before the protocol amendment and two confirmed partial responses were noted in the cohort included after the implementation of this dose adjustment policy. The rate of tumour control (partial plus minor responses plus stable disease) was 38.5% (5/13) *vs* 50% (7/14) in those patients who received trabectedin before and after the amendment, respectively.

### Pharmacokinetics

Blood samples were collected and analysed in 10 cases. First- and second-cycle PKs were assessed in seven patients, whereas in three patients, PKs were assessed only at the first cycle. Table 4 shows the PK parameters at the first and second cycles. The results are fully consistent with PK data from the previous phase I study (Van Kesteren *et al*, 2000)

## DISCUSSION

The success of alternative chemotherapy in patients with metastatic breast cancer who have failed after previous therapy with anthracyclines or taxanes has been modest. Moreover, the selection of the most beneficial salvage regimen is becoming a major concern because of the widespread use of these drugs as first-line chemotherapy. Therefore, the identification of new active agents feasible for chronic treatment and suitable for combination therapy is a priority.

The present study has shown that trabectedin is active in previously treated breast cancer, inducing objective responses and tumour control. The partial response rate (14%), although modest, is within the range of what can be expected from an active drug in this setting and is along the lines of the findings in a recent phase II study (Gurtler *et al*, 2005), comparing two dose schedules of trabectedin in pretreated breast cancer. The authors reported a 13% partial response rate with 1.3 mg m^−2^ as a 3-h i.v. infusion every 3 weeks, whereas a weekly lower dose was less active. Moreover, in the present study, two patients had a confirmed minor response and six had stable disease. The overall survival also compares well with other salvage regimens. Evidence of biological activity, as evidenced by drops in tumour markers, was observed in eight patients.

Neutropenia and transaminitis were the most commonly observed drug-related toxicities. Episodes of neutropenia were transient and no episodes of neutropenic fever occurred. Although transaminitis occurred in the majority of patients, it never led to serious adverse events. Other side effects were asthenia and emesis, which were controllable by corticoids and conventional antiemetic therapy. The absence of other severe toxicities, including grade 3–4 alopecia, and the lack of cumulative toxicities make trabectedin feasible and attractive for long-term palliative use.

In addition to the activity reported for trabectedin as a single agent, mechanistic (D'Incalci *et al*, 2002; Kanzaki *et al*, 2002) and preclinical (Takahashi *et al*, 2002; D'Incalci *et al*, 2003; Meco *et al*, 2003) studies have noted evidence of *in vitro* and *in vivo* synergism of trabectedin in combination with anthracyclines, taxanes and platin salts in experimental models of several solid tumours, including breast cancer. Phase I combination studies have also reported feasibility and activity in resistant solid tumours when trabectedin is combined with anthracyclines (Gianni *et al*, 2003; Cohen *et al*, 2005), paclitaxel (Chu *et al*, 2004), platinum compounds (Sessa *et al*, 2004) or capecitabine (Holden *et al*, 2003). The final results of these phase I studies of trabectedin combinations will allow us to select the regimen with the best therapeutic profile for further phase II and potential phase III trials in previously treated breast cancer. The PK data generated in this small cohort of patients appears to be consistent with the overall PK profile of the compound. With the major caveat of the limited evidence generated, the comparative PKs on first *vs* second course suggest similar area under the curve and terminal half-life figures but a lower *C*_max_ value on the second course of therapy. Ongoing population PK analysis in large cohorts of cases treated with trabectedin will provide soon with valuable information on the PK/pharmacodynamic profile of this compound. In any case, our results confirm that plasma levels well above the active *in vitro* concentrations are reachable and sustainable at dose level that is feasible for multiple cycles. Such findings, essentially the long terminal half-life of trabectedin, also support the incorporation of the 3 h i.v. infusion at least in breast cancer, where the rate of objective response with such short infusion schedule (Gurtler *et al*, 2005) is identical to the one reported in this study.

A 3-h i.v. infusion has been included in all the trabectedin-containing combination regimens.

Trabectedin is a DNA-interacting agent that requires a functional transcription-coupled nucleotide excision repair (TC-NER) DNA repair system to induce antiproliferative effects in experimental cancer models (Takebayashi *et al*, 2001; D'Incalci *et al*, 2003). A retrospective analysis (Van Oosterom *et al*, 2004) examining polymorphisms and mRNA expression levels of genes of the TC-NER and the homologous recombination repair (HRR) DNA repair pathways in 45 advanced sarcoma patients treated with trabectedin reported a better outcome in patients bearing a functional TC-NER pathway. Moreover, low levels of BRCA1, which is involved in HRR, correlated with a better response rate, time to progression (*P*=0.06) and median survival (*P*=0.02). Human sarcoma primary cultures with mutated p53 have also shown extreme *in vitro* sensitivity to trabectedin (A Carnero, personal communication). These pharmacogenomic findings merit investigation in breast cancer patients treated with trabectedin. By elucidating the molecular basis behind individual differences in outcome, we will be able to design customised models for therapeutic intervention and maximise the clinical impact of trabectedin in patients with advanced pretreated breast cancer.

## Figures and Tables

**Figure 1:**
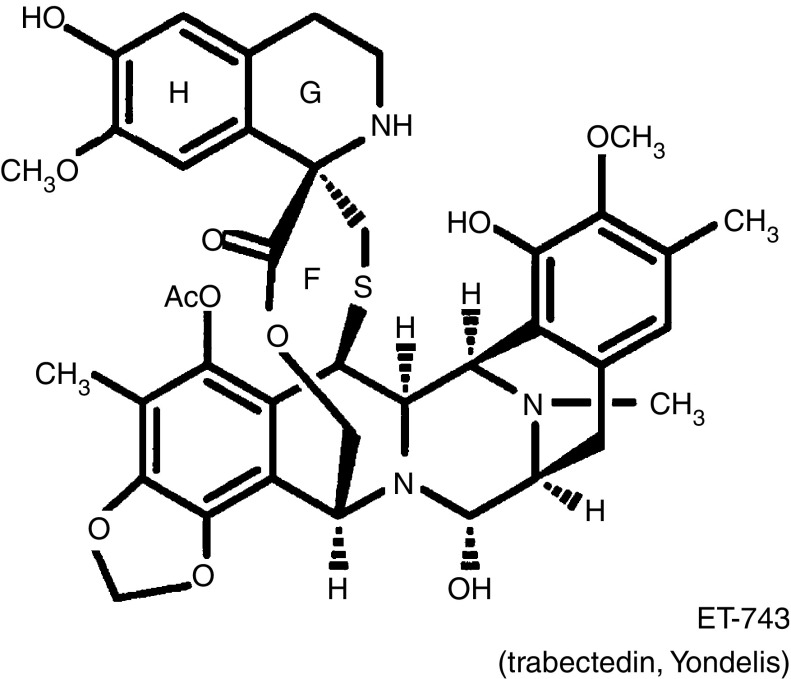
Chemical structure of trabectedin.

**Table 1:** Patient characteristics

**Parameter**	**Number**
Median age (range)	54 years (37–67)
	
*ECOG PS at entry*	
0	5
1	22
Median number of sites (range)	2 (1–5)
	
*Site of disease*	
Liver	16
Lung	10
Bone	12
Nodes	11
Breast	6
Skin	5
Pleura	2
	
*Previous therapies*	
Surgery	27
Radiotherapy	24
	
Chemotherapy	
Median number of previous lines (range)	2 (1–4)
Median number of previous agents (range)	5 (2–7)
	
*Previous exposure*	
Anthracyclines	27
Taxanes	23

PS=performance status.

**Table 2:** Trabectedin: characteristics of patients with objective response

**Age**	**PS**	**Prior CT lines^a^**	**Prior CT agents**	**Anthra clinical resistance**	**Tax clinical resistance**	**Site of disease**	**OR**	**Number of cycles**	**Baseline CA 15.3**	**CA 15.3 Maximal nadir**	**TTP**
51	0	1+1	5	E	E	Lung, mediastinal nodes	PR	4	26	7	3.0
53	0	2+2	5	E	NA	Skin, mediastinal nodes	PR	12	30	25	11.3
57	1	1+1	4	E	E	Lung, liver	PR	10	1061	125	7.3
46	0	1+2	5	E	R	Breast, axillary & peritoneal nodes	UPR	4	1604	1425	3.5
55	1	1+1	5	E	E	Liver	MR	5	197	88	5.0
53	0	0+3	7	E	E	Lung, pre-external soft tissue	MR	4	32	29	6.0

a Adjuvant+advanced.

Anthra=anthracyclines; CT=chemotherapy; E=exposed; MR=minor response; NA=information not available; OR=overall response; PS=performance status; R=primary resistant; Tax=taxanes; TTP=time to progression in months; U=unconfirmed.

**Table 3:** Safety profile of trabectedin in advanced pretreated breast cancer^a^

**Parameter**	**G1**	**G2**	**G3**	**G4**
ASAT	1	6	15	4
ALAT	1	5	12	8
Bilirubin	—	2	1	—
Alk-Phos	14	6	—	—
Gamma-GT	2	8	12	1
Vomiting	5	6	2	1
Diarrhea	6	—	—	—
Mucositis	1	—	—	—
Fatigue	7	12	3	1
Neutropenia	2	4	9	9
Thrombotytopenia	2	3	1	1
Anaemia	7	14	—	—

a NCIC-CTC modified criteria/worst grade per patient while on therapy. *N*=27.

**Table 4:** Pharmacokinetic analysis^a^

	**Parameter**				
**Cycle**	**AUC (h *μ*g l^−1^)**	***C*_max_** **(pg ml^−1^)**	***t*_1/2_**	**CL (l/h m^2^)**	**Vss (l m^−2^)**
First	43.76	1220.4	42.72	66	1010
Second	45.85	997	49.1	67.9	1459.6

a median values.

AUC=area under the curve; *C*_max_=maximum plasma concentration; CL=plasma clearance; *t*_1/2_=elimination half-life; Vss=volume of distribution at steady state.
